# Parental Perspectives on the Use of Smartwatch Activity Trackers by Young Children: Qualitative Study

**DOI:** 10.2196/79851

**Published:** 2025-11-11

**Authors:** Raymond J Davey, Amity Campbell, Amber Beynon, Charlotte Lund Rasmussen, Danica Hendry, Sarah Stearne, Courtenay Harris, Leon Straker, Juliana Zabatiero

**Affiliations:** 1 Curtin School of Allied Health Faculty of Health Sciences Curtin University Bentley Australia; 2 ARC Centre of Excellence for the Digital Child Perth Australia

**Keywords:** wearable activity tracker, smartwatch, preschool children, physical activity, qualitative research

## Abstract

**Background:**

Smartwatch activity trackers are devices that measure physical activity levels with features that aim to encourage physically active behaviors. These devices have shown promise for increasing physical activity levels and reducing sedentary behaviors among school-aged children, adolescents, and adults. Recently, commercially available products have been adapted so that they are suitable for use by preschool-aged children. However, it is unclear whether the intended use of these devices is feasible and effective in young children.

**Objective:**

The purpose of this study was to explore parents’ perspectives on the use of smartwatch activity trackers by young children.

**Methods:**

Semistructured interviews were conducted with 22 parents (17/22, 77% female) of children aged 3-5 years. Interviews explored perspectives on the feasibility of their children wearing the devices, implications of use by young children, and how families could make use of these devices to support their children’s physically active behaviors. Interviews were audio-recorded, transcribed verbatim, and data analyzed using thematic analysis.

**Results:**

Parents perceived that the use of these devices by young children is feasible, with developmental stage or abilities and personality or temperament being important individual determinants of feasibility. However, parents expressed concerns related to the devices providing extrinsic motivation to move, being disruptive or distracting, being a burden on parents, and for the safety and privacy of their child’s information. Most parents believed that young children are inherently active and do not need devices to support physical activity. Furthermore, most parents expressed an interest in knowing how physically active their children were and thought that there may be a role for these devices for children who are less physically active.

**Conclusions:**

Parents reported developmental stage or abilities and temperament as relevant considerations related to the feasibility of smartwatch activity tracker use by young children. Parents also indicated that there is a potential role for these devices in young, less active children.

## Introduction

Higher levels of physical activity are associated with reduced adiposity and better cognitive and motor skill development, psychosocial health, bone and skeletal health, and cardiometabolic health in preschool children [1,2]. Furthermore, in this age group, participation in physical activity helps to offset the risks of excessive sedentary behavior, with higher levels of sitting associated with increased adiposity and reduced psychosocial health and cognitive development [3,4]. Establishing physically active behaviors at this age also influences engagement in physical activity throughout childhood, adolescence, and adulthood [5].

International health guidelines recommend that preschool children (aged 3 to 5 years) spend at least 180 minutes per day in a variety of physical activities at any intensity, of which, at least 60 minutes is moderate to vigorous physical activity [6,7]. In addition, replacing restrained sedentary behaviors and sedentary screen time with physical activity is suggested to provide additional health benefits [6,7]. Unfortunately, many preschool children in developed countries do not meet current physical activity recommendations [8-10] and spend significant proportions of their waking time sitting or inactive [10,11]. Therefore, a current public health priority is to develop intervention programs that aim to increase physical activity levels among young children, when these formative experiences can shape future behaviors.

Smartwatch activity trackers are wrist-worn devices that incorporate accelerometers to provide measures of physical activity such as the number of steps taken, distance travelled, time spent active or inactive, and the intensity of activity. This information is typically shared with the user via a small digital interface on the wearable device itself and additionally via synchronization to mobile or web-based apps that collect and summarize the physical activity data. These devices also include features that aim to encourage physically active behaviors and disrupt sedentary behaviors. For instance, users can set daily physical activity goals and receive notifications about their progress. These devices have been shown to provide valid and reliable estimates of physical activity in children [12]. They have also shown promise for increasing physical activity levels and reducing sedentary behaviors among school-aged children, adolescents, and adults [13,14]. Specifically, interventions that incorporate these activity trackers have shown small to moderate favorable effects on physical activity minutes per day, steps per day, and moderate to vigorous physical activity levels in children and adults [13-17]. However, it is unknown whether smartwatch activity trackers can have similarly favorable effects on physical activity in preschool children. In a recent systematic review, we showed that while the use of these devices may be feasible in preschool children, to date, no studies have evaluated their efficacy for increasing physical activity in this age group [18].

With the increase in commercially available smartwatch activity trackers designed for use in preschool-aged children, evidence is needed on the feasibility and efficacy of influencing the physical activity of young children. Given the important role parents would have in mediating the use of this technology by preschool children, this study aimed to explore parents’ perspectives on the feasibility of young children wearing activity trackers, potential implications of young children using these devices, such as advantages, disadvantages, and unintended consequences of use, and possible roles for activity trackers in this age group.

## Methods

The description of the methodology follows established Standards for Reporting Qualitative Research [19].

### Study Design

This qualitative study used single time-point semistructured interviews, following an exploratory, cross-sectional, observational design [20]. An interpretive description approach was chosen, where the perceptions and experiences of the participants were explored by analyzing concepts, themes, and patterns in the interview data, with the view to generating practically useful outcomes [21]. This follows a constructivist or interpretivist paradigm, with our rationale being that the perspectives and experiences of our participants are necessary to meet our study aims. This approach may limit the transferability of our research findings to people in different contexts to those of the participants of this study.

### Participants

The participants were parents or primary caregivers of children aged 3 to 5 years. Parents or primary caregivers were defined as the primary person providing support to the child over a long period of time and involved in daily care routines such as feeding, hygiene, play, sleep, or health. Participants were recruited through purposive and snowball sampling from existing networks of the Curtin Healthy Digital Child research team including the networks of their community advisory groups [22]. In addition, recruitment flyers were distributed nationally using the Australian Research Council Centre of Excellence for the Digital Child social media [23]. The recruitment material provided links to the participant information statement and consent form. Once informed consent was obtained, the participants were provided with a link to an online preinterview questionnaire (Qualtrics XM). Following this, the participants completed an interview either in person or online using videoconferencing technology.

### Preinterview Questionnaire

The preinterview questionnaire (Multimedia Appendix 1) included questions on sociodemographic characteristics (eg, age of participant and age and sex of child, number of children, suburb of residence, occupation, and highest education level). This information was used to tailor each participant’s semistructured interview and support data analysis.

### Qualitative Semistructured Interview

The interviews followed a semistructured format guided by open-ended questions to allow parents to express a broad range of perspectives openly and honestly without their responses being directed by the question itself. The researcher (RJD) who conducted the interviews is the father of a preschool child with a background in health research and education. Although the questions were designed to allow free expression, it is possible that these interviewer characteristics may have introduced biases or influenced the relationship with the participants through shared experiences. The interview questions related to the use of wrist-worn smartwatch activity trackers by 3- to 5-year-old children. In particular, the questions explored their perspectives on the feasibility of young children wearing these devices, the potential implications of young children using these devices, and the potential roles or applications for these devices in the lives of young children. The questions were developed by a multidisciplinary team of researchers in consultation with the Curtin Healthy Digital Child Parent and Professional Advisory Groups. Each question was mapped to at least one of the three themes directly aligned with the research aims: (1) feasibility, (2) implications of use, and (3) potential roles. The average duration of interviews was 33 (SD 8; range 20-53) minutes. Each interview was audio-recorded (with consent), and the recordings were professionally transcribed verbatim.

Feasibility has been defined as the extent to which the devices are suitable for the target population, can be successfully delivered to the target population, or show promise of being successful within the intended population [12]. Based on this definition, the following open-ended questions were posed to the participants to capture their perceptions of the feasibility of activity tracker use by young children: Does your child use an activity tracker? Do you think young children could use these devices? Do you see a role for activity trackers in your child’s life?

Implications were broadly defined as any advantages, disadvantages, or unintended consequences of use that may or may not be related to the device’s primary purpose of physical activity monitoring. The open-ended questions that were posed to the participants to capture their perceptions of the implications of activity tracker use by young children included the following: What issues do you think parents might raise? Can you think of any other considerations for young children using activity trackers?

Potential roles included any application for smartwatch activity trackers identified by the participants in either their child’s life or the lives of other 3- to 5-year-old children. The open-ended questions that were posed to the participants to capture these potential roles included the following: Do you see a role for activity trackers in your child’s life? Do you think parents have a good sense of how much physical activity their child does? Would you be interested to know?

### Data Analysis

Data from the preinterview questionnaire were analyzed using descriptive statistics, reported as counts and percentages, to provide a summary of participants’ characteristics. The audio recordings of the open-ended questions were transcribed verbatim, and transcriptions were then analyzed by inductive thematic analysis [24] using NVivo (Lumivero). Data collection and analysis were conducted simultaneously to allow the research team to respond to evolving study findings. This iterative process also allowed the research team to identify saturation of emergent themes [22], which informed the decision to stop recruitment and data collection naturally.

### Ethical Considerations

This study was approved by the Curtin University Human Research Ethics Office (approval: HRE2023-0608). Prospective participants were provided with a detailed information sheet describing the purpose of the study; the benefits, risks, and expected outcomes of the study; and the research team. Following this, the participants provided informed consent before completing a questionnaire, and an interview was conducted either in person or using secure videoconferencing software. At the beginning of each interview, the participants were asked to provide verbal confirmation of their consent for the interview to be audio-recorded. The interview questions were not sensitive in nature and open-ended to allow free expression of perspectives. The participants were reminded that they were not obliged to answer the questions and that they could stop the interview at any stage. To ensure participant privacy, the data collected were either deidentified or coded and stored on secure Curtin University research drives, accessible by the research team only. The participants were not paid for their involvement in the study but were offered a US $32.7 gift card for their time.

## Results

### Participant Characteristics

The participant characteristics are presented in Table 1. In total, 22 parents aged 31 to 43 years (mean 36.5, SD 3.6 years) of children aged 3 to 5 years, inclusive (mean 4.3, SD 0.9 years) completed this study. This included 17 mothers and 5 fathers of 1 to 3 (mean 2.1, SD 0.6) children. The most common occupations were health professionals (5/22, 23%), academics (4/22, 18%), clerical and administrative workers (3/22, 14%), education professionals (3/22, 14%), and stay-at-home parents (3/22, 14%), with 18/22 (82%) who had completed tertiary education. Most participants lived in Western Australia (16/22, 73%), with others located in Victoria (3/22, 14%), New South Wales (2/22, 9%), and South Australia (1/22, 5%).

**Table 1:** Participant characteristics (N=22).

Characteristic			Values, n (%)
**Age (years)**			
	18-24	0 (0)	
	25-34	8 (36)	
	35-44	14 (64)	
	>45	0 (0)	
**Sex**			
	Female	17 (77)	
	Male	5 (23)	
**Children**			
	1 child	3 (14)	
	2 children	13 (59)	
	3 children	6 (27)	
**Age of child (years)**			
	3 to <4	10 (45)	
	4 to <5	7 (32)	
	5 to <6	5 (23)	
**Occupation**			
	Manager	1 (5)	
	Academic	4 (18)	
	Health professional	5 (23)	
	Education professional	3 (14)	
	Social and welfare professional	2 (9)	
	Clerical and administrative worker	3 (14)	
	Stay-at-home parent	3 (14)	
	Defense	1 (5)	
**Highest education level**			
	High school or equivalent	1 (5)	
	Postsecondary, nontertiary	3 (14)	
	Bachelor degree or equivalent	7 (32)	
	Postgraduate degree	11 (50)	
**Location**			
	Western Australia	16 (73)	
	New South Wales	2 (9)	
	Victoria	3 (14)	
	South Australia	1 (5)	

### Feasibility

#### Overview

When parents considered the feasibility of smartwatch activity tracker use by young children, the following subthemes were identified: age and developmental abilities, personality and temperament, capacity for independent use, novelty and maintenance of use, and wearability. Supporting quotes for each of the subthemes are presented in Table 2.

**Table 2:** Parental perspectives on the feasibility of smartwatch activity tracker use by young children.

Subthemes	Supporting quotes
Age and developmental abilities (17/22, 77%)	“I think there’s a big difference between a three-year-old and a five-year-old” [Participant 2, mother of 3 children including a 3-year-old]. “I think definitely a five-year-old would probably be more engaged and have more of an understanding of the device” [Participant 2, mother of 3 children including a 3-year-old]. “... as a three-year-old, I just don’t think that she would make the link yet” [Participant 2, mother of 3 children including a 3-year-old]. “I think for [my 5-year-old child] I think she’d definitely be capable in appreciating—I guess engaging with it, and I think that she would interact with it and understand and equate the game with moving more. [My 3-year-old child] I’m not so sure about just because of his age. I don’t think [my 3-year-old-child] would be at that level to make that connection” [Participant 3, father of 3 children including a 3-year-old and a 5-year-old].
Personality and temperament (16/22, 73%)	“... it would depend on the child and what motivates them” [Participant 21, mother of 2 children including a 4-year-old]. “... he has that kind of personality where he responds really well to challenges or to goals” [Participant 1, mother of 2 children including a 5-year-old]. “I think for kids that are really rewards-focused or goal-oriented, it could be really valuable to them to motivate them to do a bit more” [Participant 1, mother of 2 children including a 5-year-old].
Capacity for independent use (21/22, 95%)	“I feel like he would need quite a bit of help still ... I think at this age; you’d probably need to make good use of those incentives and those rewards and walk him through that. And then once he gets the hang of it, I think he’d probably be okay, but it would take a bit of work” [Participant 11, mother of a 3-year-old child]. “I think it would be pretty led by the parents. I think they’re probably still too young to do it independently, I would think” [Participant 7, mother of 2 children including a 5-year-old].
Novelty and maintenance of use (15/22, 68%)	“... maybe initially they would find it really exciting and they would use it like a toy, and then I think the novelty would probably wear off if they weren’t that engaged with it and it ... prompted them to do something and they didn’t want to, it would become a chore and not a game anymore” [Participant 15, mother of 2 children including a 4-year-old]. “I wonder whether the novelty of it would wear quickly, so I’m not sure how long-term it would make changes for either parents or as a family unit” [Participant 15, mother of 2 children including a 4-year-old].
Wearability (11/22, 50%)	“I think the biggest thing would be keeping it on them and having them wear it all the time. Because even just getting her to keep shoes on can be a bit of a hassle, so I imagine something that’s around her wrist, I think that she wouldn’t necessarily want to keep on” [Participant 18, mother of 2 children including a 3-year-old]. “I think that actually wearing it would be the biggest hurdle for her” [Participant 18, mother of 2 children including a 3-year-old]. “... she might find wearing a watch to be uncomfortable or irritating” [Participant 2, mother of 3 children including a 3-year-old]. “... going in the sandpit, sand caught under it, hot and sweaty in summer ... not tolerated from a sensory perspective” [Participant 2, mother of 3 children including a 3-year-old].

#### Subtheme: Age and Developmental Abilities

This subtheme relates to perspectives that 3 to 5 years is a developmentally diverse age range with children at different stages of cognitive development and having varied literacy, understanding of the purpose or intention of the device (including the rewards and prompts), and abilities to respond to it. Most parents (17/22, 77%) reported this as a key determinant of feasibility.

#### Subtheme: Personality and Temperament

This subtheme relates to perspectives that individual differences would influence the uptake and maintenance of use of these devices. Most parents (16/22, 73%) reported that these devices would suit children who are goal- or reward-focused, competitive, or compliant.

#### Subtheme: Capacity for Independent Use

This subtheme relates to perspectives on the capacity of children to use and engage with the device independently as intended, for example, receive and respond to information, purposefully aim to achieve movement goals, and understand and seek rewards. Almost all parents (21/22, 95%) expressed a view related to this subtheme, but their perspectives were mixed, with the child’s age, abilities, and personality being key determinants of their capacity for independent use and their need for support.

#### Subtheme: Novelty and Maintenance of Use

Many parents (15/22, 68%) reported that novelty may encourage uptake and engagement by young children, but that the maintenance of use would be limited in this age range, as interest might diminish over time.

#### Subtheme: Wearability

Half of the parents (11/22, 50%) also indicated that issues related to wearability (eg, tolerance to wear and comfort) would impact the feasibility of use of these devices by their children.

### Implications

#### Overview

When considering the implications of smartwatch activity tracker use by young children, the following subthemes were identified: the potential negative influence of extrinsically motivating physical activity, disrupting positive behaviors, the burden on parents, and the safety and privacy of information. Supporting quotes for each of the subthemes are presented in Table 3.

**Table 3:** Parental perspectives on potential implications of smartwatch activity tracker use by young children.

Subthemes	Supporting quotes
Extrinsically motivating physical activity (17/22, 77%)	“... kids would then place emphasis on rewards for activity rather than just enjoying and initiating activity of their own accord” [Participant 14, mother of 2 children including a 5-year-old]. “I think the games are a good motivator, but whether or not this is going to affect their overall intrinsic motivation for exercise in the long term, I’m not sure” [Participant 2, mother of 3 children including a 3-year-old]. “I like the idea that it can help foster better physical activity, but I just wonder whether it comes at the expense of driving an implicit and internal motivation to engage in exercise, whether it’s going to the playground for enjoyment, engaging with friends, playing sport” [Participant 3, father of 3 children including a 3-year-old and a 5-year-old]. “I’d like them to engage in physical activity and exercise because they enjoy the activity, not because they’re motivated by what’s on their wrist” [Participant 3, father of 3 children including a 3-year-old and a 5-year-old].
Disrupting positive behaviors (16/22, 73%)	“Will they stop what they are currently so intently focused on to do what this thing is telling them to do?” [Participant 13, mother of 2 children including a 3-year-old]. “... these ones are very visible, so that sense of being almost a distraction at times, or intrusive to what’s going on in the present” [Participant 6, father of 3 children including a 3-year-old and a 5-year-old]. “I do have concerns about the interactive nature of it ... as much as they would use them, I wouldn’t want them to overuse them” [Participant 12, mother of 3 children including a 3-year-old and a 4-year-old]. “I do fear that it would be more a case of them sitting on the lounge looking at their watch, rather than actually getting up and doing stuff” [Participant 12, mother of 3 children including a 3-year-old and a 4-year-old].
The burden on parents (18/22, 82%)	“... has the potential to just be another source of guilt that we’re not doing enough as parents” [Participant 10, mother of 2 children including a 4-year-old]. “... you’re adding to parents’ burden and that feeling that we’re never doing enough” [Participant 12, mother of 3 children including a 3-year-old and a 4-year-old]. “I think if the information goes to the parent or the parent is managing that feedback, it would be up to the parent to be careful in the way they—what they do with that information in relation to the child and how much information they want to share with their child especially if there’s a need for improvement” [Participant 11, mother of a 3-year-old child].
Safety and privacy of information (14/22, 64%)	“Can people find out where my child is or what they’re doing?” [Participant 16, mother of 2 children including a 4-year-old]. “... some parents would be concerned about the tracking of their children’s movements and whether or not that could be hacked” [Participant 18, mother of 2 children including a 3-year-old]. “I think maybe the privacy stuff around the data and how secure that is, and where it goes and what it’s used for” [Participant 6, father of 3 children including a 3-year-old and a 5-year-old].

#### Subtheme: Extrinsically Motivating Physical Activity

This subtheme relates to perspectives that activity trackers are a source of extrinsic motivation (eg, rewarding achievement) that may displace children’s intrinsic motivation to move simply for fun and enjoyment. Most parents (17/22, 77%) reported this implication of use.

#### Subtheme: Disrupting Positive Behaviors

This subtheme relates to perspectives that the device may be intrusive and disrupt children from positive behaviors including physical activity itself, as well as immersive, creative play, or rest. Most parents (16/22, 73%) reported this concern.

#### Subtheme: The Burden on Parents

Most parents (18/22, 82%) expressed the concern that the use of these devices by young children would impose a burden on parents because of their essential role in interpreting and delivering the information from the devices to their children, together with the guilt parents may feel when recommendations or goals are not met.

#### Subtheme: The Safety and Privacy of Information

Two-thirds of parents (14/22, 64%) indicated that safety and privacy of information is a concern, primarily related to the security of identifiable data and location information as well as the possibility of companies gathering information about children’s behaviors and lives.

### Potential Roles

#### Overview

When the possible roles for smartwatch activity trackers for young children were considered, the following subthemes were identified: a limited need for a device to encourage physical activity in young children because they are inherently active; benign activity tracking to measure, but not influence, physical activity levels to improve parents’ awareness of their child’s physical activity behaviors; creating opportunities to discuss physical activity in a family setting; as a means to encourage physical activity in children who are less active, and as a supporting tool that is part of a broader program to increase physical activity levels in young children. Supporting quotes for each of the subthemes are presented in Table 4.

**Table 4:** Parental perspectives on the potential roles of smartwatch activity tracker use by young children.

Subthemes	Supporting quotes
Limited—young children are inherently active (20/22, 91%)	“... she’s motivated to move her body regardless of the activity tracker or not. I think intrinsically as a three-year-old, she does a lot of activity without even thinking about it or without even really knowing, comprehending what it is and what it means” [Participant 2, mother of 3 children including a 3-year-old]. “... it wouldn’t ever cross my mind that a typical preschooler would need one only because of how much my own child loves sport and movement already. It’s hard to get him to rest” [Participant 11, mother of a 3-year-old child]. “I’m not too concerned because I just have a general impression that she’s busy and she’s active and she’s enthusiastic and she’s happy to be active when we are. So, I’m not particularly worried” [Participant 5, mother of 3 children including a 3-year-old].
Increase awareness of physical activity levels (20/22, 91%)	“I would be super interested to ... see how much he actually does” [Participant 1, mother of 2 children including a 5-year-old]. “... it would be interesting ... to know how much (her child) is doing and when he’s active, maybe the circumstances” [Participant 1, mother of 2 children including a 5-year-old]. “Yeah, so it would be interesting to know how much she really is moving during the day” [Participant 14, mother of 2 children including a 5-year-old]. “I would definitely be interested to know exactly how much physical activity she’s doing and when, and then how that might potentially affect other things like sleep and things like that” [Participant 2, mother of 3 children including a 3-year-old].
Opportunities to discuss physical activity (13/22, 59%)	“You can have little discussions with them about, what did you do today? Did you feel puffed afterwards? Was it fun?” [Participant 4, mother of 2 children including a 3-year-old]. “How many steps have I done? And how many steps have you done? And what’s yours saying? And what’s mine saying?’ So, that social element of it I think would bring more interest, rather than it being a personal thing” [Participant 6, father of 3 children including a 3-year-old and a 5-year-old].
Special circumstances (17/22, 77%)	“I think that activity trackers could play a role for those sorts of kids who don’t or don’t have an interest in being active” [Participant 3, father of 3 children including a 3-year-old and a 5-year-old]. “I mean, maybe if there was an obvious problem and the specific child didn’t get enough physical activity, or they were really against going to the park and doing all those things” [Participant 2, mother of 3 children including a 3-year-old]. “I think if I was worried that I had a really sedentary kid ...” [Participant 5, mother of 3 children including a 3-year-old]. “I can imagine that if you lived in a flat or an apartment and you only had a very small space and not much access to outdoors, you might also be worried” [Participant 5, mother of 3 children including a 3-year-old]. “I think there are plenty of situations where these trackers would be beneficial and would potentially help kids and families [without adequate] education, knowledge, and background [related to the benefits of physical activity to health]” [Participant 10, mother of 2 children including a 4-year-old].
Part of a broader program (10/22, 45%)	“I can see that (it) would be another tool in their belt on how to help a family” [Participant 15, mother of 2 children including a 4-year-old]. “I think it’s probably best to have a range of approaches. So this could be one of a package of—if you’re looking to increase activity in young children, have this as part of a broader package, not the only thing that we’re relying on” [Participant 14, mother of 2 children including a 5-year-old].

#### Subtheme: Limited—Young Children Are Inherently Active

This subtheme relates to perspectives that there may not be a role for these devices in this age range because young children are naturally physically active without encouragement or support and parents are generally not concerned about their children’s physical activity levels. Almost all parents (20/22, 91%) reported this view.

#### Subtheme: Increase Awareness of Physical Activity Levels

This subtheme relates to perspectives that there may be a role for these devices to support parents’ awareness of their children’s physical activity levels and the settings or circumstances that either facilitated or constrained this behavior. Almost all parents (20/22, 91%) reported that they would be interested in using these devices for this purpose.

#### Subtheme: Opportunities to Discuss Physical Activity

This subtheme relates to perspectives that the use of activity trackers may facilitate discussions about physical activity between parents and their children. Some parents (13/22, 59%) indicated that there may be a role for activity trackers to prompt conversations about physical activity with their children to both encourage the behavior and educate their children on its benefits.

#### Subtheme: Special Circumstances

This subtheme relates to possible applications for activity trackers in circumstances where the ability or opportunity to participate in physical activity may be limited. Most parents (17/22, 77%) could see a potential benefit in instances where children are not getting enough physical activity due to health issues, a natural inclination to be physically inactive, or other family, social, or environmental factors.

#### Subtheme: Part of a Broader Program

Almost half of the parents (10/22, 45%) suggested that there may be an application for activity trackers as an adjunct or supporting strategy to encourage physical activity levels but not the sole strategy.

## Discussion

### Principal Findings

The purpose of this study was to explore parents’ perspectives related to the feasibility, potential implications, and possible roles for smartwatch activity tracker use by young children. While the parents in this study perceived the use of smartwatch activity trackers by young children as feasible, the potential negative influence of extrinsic motivation to physical activity, disruption of positive behaviors, parental burden on parents to mediate the information from the device, and the safety and privacy of their child’s information are important implications to consider. These implications, together with the pervasive view that young children are naturally physically active, could explain why these parents did not see a broad role for smartwatch activity trackers to promote physical activity in this age group. However, these parents indicated that there may be a role for the use of these devices to increase parents’ awareness of their child’s physical activity levels and patterns, to create opportunities to discuss physical activity, and in specific circumstances when children may not be getting enough physical activity for individual or family or social or environmental reasons.

### Feasibility

The overarching view from parents was that the use of smartwatch activity trackers by young children is feasible. This view is supported by findings of recent systematic reviews that report 80% to 100% adherence to wear by young users (aged 3-7 years) as a proxy measure of feasibility [12,18]. However, parents reported that child characteristics related to their age, developmental abilities, and temperament, as well as the potential need for parental support to use the device and interpret its information, are likely to be key determinants of uptake and ongoing use by young children. Aligned with these perceptions is the observation that a sense of competence and self-efficacy are drivers of engagement in physical activity [25]. This could explain why individual factors that influence this sense of competence, including age or development or abilities and personality or temperament, are key in determining the feasibility of activity tracker use by young children. Smartwatch activity trackers incorporate behavior change techniques, including goal setting, action planning, and behavioral practice or rehearsal, which have been developed based on frameworks derived from theories of adolescent or adult motivation. Although such frameworks may not be the most relevant for young children, they have shown some promise in interventions to increase physical activity in children younger than 6 years of age [26]. However, the degree to which these devices influence a sense of competence and other key tenets of self-determination theory that support motivation for physical activity may depend on individual factors in addition to developmental stage [25]. For instance, goal setting and self-monitoring may support competence and self-efficacy in some children [27-29] but perhaps not in others who do not place as much value on goal achievement. Furthermore, parent involvement and support may be important for promoting relatedness, which also underpins intrinsic motivation for physical activity behaviors [27,30]. This may be particularly relevant for younger users who rely on the involvement of their parents.

Parents also indicated that the novelty of using an activity tracker may encourage uptake initially, but ongoing use may be limited in this age range. This view is consistent with findings in older children (aged 5 to 19 years), where adherence to wear declines after 2 to 4 weeks of use [31]. Poor maintenance of activity tracker use over time has also been reported in young children [18]. Furthermore, the view that comfort and wearability would also influence feasibility has been previously reported by parents of preschool children [32,33] and is corroborated by studies in young children [18] and older children (aged 5 to 19 years) [31,34]. This may be an important consideration for practical applications including intervention studies.

### Implications

While parents in this study could see the positive intention of the devices to encourage or support physically active behaviors, they also raised some relevant considerations about this approach. These included that the device may be a source of extrinsic motivation for physical activity that could displace intrinsic desires to simply move and play for fun and enjoyment. Specifically, many devices include certain features designed to motivate movement (eg, prompts or reminders to move, daily physical activity goals with updates on progress, and rewards for achieving certain milestones or targets). In support of this implication raised by the parents, extrinsic rewards for completing tasks or activities have been shown to reduce subsequent intrinsic interest and motivation for these behaviors in young children [35,36]. Furthermore, intrinsic motivation has been shown to be the only type of motivation that is associated with physical activity behavior in children, and it has been recommended that interventions to increase physical activity in children should aim to optimize enjoyment and inherent satisfaction in being active rather than relying on forms of extrinsic motivation [37]. Thus, the extrinsic motivation theory basis for activity trackers may not be appropriate for young children. Additionally, the potential for the feedback from the device to be misinterpreted or create feelings of pressure to achieve physical activity goals or guilt associated with not meeting goals has been reported in school-aged children and adolescents [38]. Relatedly, some parents indicated that how the information from the activity trackers was interpreted by young children has the potential to shape their involvement in physical activity (eg, children interpret low numbers or missed goals as failures, not being good enough, or being naughty). However, the potential negative effect of smartwatch activity trackers on intrinsic motivation to be active and feelings of pressure or guilt associated with meeting goals have not been specifically investigated in young children.

Parents also indicated that the devices could disrupt or distract positive behaviors, with the potential to interrupt learning or immersive play. This perception may be related to the importance that parents place on opportunities for immersive, enriching play that contributes to learning, socialization, and emotional and physical development [39,40]. It may also reflect parents’ reluctance to overuse technology. It has been reported that parents have mixed perceptions of young children engaging with digital technologies with both perceived benefits (eg, supporting learning and education) and risks (eg, detrimental to cognitive and social development) [41]. It is possible that parents may be reluctant to use these devices if they do not perceive a clear benefit to their use. This possibility is supported by the parents’ view that young children are naturally active and do not need a device to support physical activity behaviors.

Another commonly reported implication was that the information provided by the devices is a potential burden on parents since they see the mediation and messaging of this information as their responsibility, and this information may also be a source of guilt for parents in instances where their child’s physical activity targets or recommendations are not met. Parents believe that in younger users, they would play a key role in interpreting and delivering feedback to the child and that they have a responsibility to deliver this information in a manner that is positive and encouraging. Some parents suggested that the opportunity for parents to present the feedback positively (eg, “let’s go outside for a walk”) or negatively (eg, “you have not met your goals, you need to do more”) would influence the child’s engagement in physical activity. Given that parents are integral to shaping early childhood physical activity behaviors [42], how parents respond to and deliver this information to their child has the potential to affect the child’s sense of achievement, self-worth, and relationship with physical activity. Activity tracker use could therefore have unintended consequences if parental framing of the information provided is not helpful. When reflecting on the intention of the smartwatch activity trackers to promote physical activity, some parents indicated that they see this as their role through modeling, providing opportunities, and expressing family values. This perspective is supported by studies that show strong associations between parental support of physical activity and their child’s participation in physical activity [43,44]. Parents believe that this role is particularly important in this age range, given the limited autonomy of young children (eg, ability to control their environment). In agreement with this view is the observation that the relationship between physical activity levels of parents and their children is strongest when their children are preschool-aged [45].

### Potential Roles

The perception by parents that young children are naturally active is consistent with previous findings and could explain why parents in this and other studies have reported that their children are sufficiently active, and they are not concerned about their children’s physical activity levels [33,46-49]. However, this view is not supported by population studies that show that many preschool children in developed countries do not meet current physical activity guidelines [8-10]. It is possible that parents overestimate the physical activity levels of their children compared to objective measures of physical activity, which has been reported in parents of less active children [49,50]. However, it has also been reported that while young children are perceived to be sufficiently active overall, their levels of moderate to vigorous physical activity may be low [48]. This observation is consistent with objectively measured physical activity levels in preschool children [51]. In this study, all children aged 3 to 5 years met the recommended 180 minutes per day of total daily physical activity, but only one-third of children met the 60 minutes per day of energetic play component of the guideline [51]. This could explain the incongruence between parents’ perceptions of their children’s physical activity levels and population data and suggests that strategies to support young children to achieve 60 minutes of energetic play per day are needed. While there may be a role for smartwatch activity trackers to support this goal, this remains to be investigated.

Despite the view that young children do not need a device to support their engagement in physical activity, many parents indicated that they would be interested to know how and when their children were physically active and that there may be a role for smartwatch activity trackers to increase their awareness of their children’s physical activity. This awareness of their child’s physical activity could inform decisions about their child’s physical activity as well as challenge the perception that increasing physical activity requires significant effort and instead can occur with small changes with little or no preparation. Thus, there could be a role for smartwatch activity tracker use in changing attitudes toward physical activity and helping to identify opportunities for increasing physical activity [52]. However, as discussed previously, some parents indicated that how the information was received by parents and delivered to children would shape the potential benefit of this opportunity. Furthermore, some parents indicated that there may be a role for smartwatch activity trackers to prompt conversations about physical activity with their children to both encourage the behavior and educate their children on the benefits of this behavior. In support of this view, it has been reported that short-term use of smartwatch activity trackers in families of preschool-aged children may encourage physical activity by prompting discussions about physical activity [52].

The perspective of most parents that the use of these devices may be beneficial for increasing physical activity levels in physically inactive children is supported by previous findings in school-aged children where programs that incorporated wearable activity trackers were more effective in inactive children [13]. Some parents indicated that smartwatch activity trackers may have a role as a supplementary tool in a toolkit of resources that encourage physical activity or address inactivity in children. These parents suggested that smartwatch activity trackers could be used as an adjunct or supporting strategy to encourage physical activity but not the only strategy. This may reflect the importance of mediating how the information the devices capture is received and interpreted by the children. Programs that incorporate wearable activity trackers in school-aged children have been shown to have a greater effect on physical activity when complementary strategies are used [13].

### Limitations

The participants included a high representation of well-educated parents in health and education professions located in Australia (primarily Western Australia) with access to parks and gardens, low-density living, and a favorable climate for engaging in outdoor physical activities. For these reasons, the perspectives reported in this study may not be transferable to other socioeconomic and geographical contexts.

### Conclusions

For most parents, the potentially negative implications of the use of these devices, together with the pervasive view that most young children are inherently physically active, meant that they see a limited role for the use of smartwatch activity trackers for supporting physically active behaviors in active young children. However, parents indicated that there may be a role for these devices to increase awareness of and promote discussions about physical activity, as well as encourage physical activity in children who are less active.

## Appendix

Multimedia Appendix 1 Preinterview questionnaire.

## Data Availability

The data generated or analyzed during this study are available from the corresponding author upon request.
