# Supplementary material for: Parental Perspectives on the Use of Smartwatch Activity Trackers by Young Children: Qualitative Study
Source: JMIR Pediatr Parent. 2025 Nov 11;8:e79851. doi: 10.2196/79851 (PMC12648127; doi:10.2196/79851)
Supplement: Multimedia Appendix 1 [file pediatrics_v8i1e79851_app1.docx]

**Project Title**

*Parental Perspectives on the Use of Smartwatch Activity Trackers by Young Children: Qualitative Study*

**Research Team Members**

*Dr. Ray Davey, Prof. Amity Campbell, Dr. Juliana Zabatiero, Prof. Leon Straker*

**Pre-Interview Survey**

1. What is your relationship to the child?
2. Mother
3. Father
4. Other (free text field)
5. What is your age?

Drop down menu: 18 – 99 years old

1. How many children do you have/care for?
2. 1 child
3. 2 children
4. 3 children
5. 4 children
6. 5 or more children
7. What is the highest level of education you have completed?
8. High school or equivalent
9. Post secondary, non-tertiary
10. Bachelor’s degree or equivalent
11. Postgraduate degree or equivalent
12. What is your occupation (including paid work, volunteer work, and/or stay-at-home parent or carer roles)?

Free text field

1. What is your family structure? Please select any that apply:
2. Married couple
3. De facto couple
4. Single parent
5. Other (free text field)
6. What is your post code?

Free text field

1. What year was your child born?

Free text field

1. What month was your child born?

Free text field
